# Attitudes towards those bereaved by a suicide: a population-based, cross-sectional study in rural Japan

**DOI:** 10.1186/1471-2458-8-334

**Published:** 2008-09-25

**Authors:** Sachiko Minamizono, Yutaka Motohashi, Masako Yamaji, Yoshihiro Kaneko

**Affiliations:** 1 Department of Public Health, Akita University School of Medicine, Akita, Japan; 2 Faculty of Health and Medicine Care, Saitama Medical University, Saitama, Japan

## Abstract

**Background:**

Family or friends bereaved by suicide are at risk of experiencing complications because of attitudes regarding suicide. It is important that individuals close to those grieving after a death by suicide demonstrate adequate knowledge and compassionate attitudes. To this end, we examined the factors that contribute to attitudes toward persons bereaved by the suicide of a family member or friend, and perceptions of suicide prevention and the promotion of mental health.

**Methods:**

A total of 5154 residents of a rural town in northern Japan aged 30–69 years completed a cross-sectional questionnaire. The questionnaire gathered data about demographic variables, depressive symptoms, and issues related to suicide including personal experience of an acquaintance's suicide, attitudes towards those bereaved by suicide, and perceptions regarding suicide prevention. Factors related to these attitudes and perceptions were analysed using logistic regression models.

**Results:**

Overall, 67.5% of respondents demonstrated appropriate attitudes towards those bereaved by suicide; 30.4% of responses were undetermined, and 2.1% were inappropriate. Undetermined attitudes were associated with male gender (adjusted OR 1.42, 95%CI = 1.26–1.61), younger age (2.64, 2.12–3.29), lower education level (1.32, 1.07–1.62), greater severity of depression (3.81, 2.80–5.20), and lack of personal experience of an acquaintance's suicide (1.39, 1.22–1.57). Inappropriate attitudes were associated with male gender (adjusted OR 1.98, 95%CI = 1.33–2.94), lower education level (2.55 1.34–4.83), and greater severity of depression (6.93, 3.52–13.67). Overall, 16.0% demonstrated passive thoughts regarding suicide prevention and the promotion of mental health in the community, and were associated with male gender (1.22, 1.04–1.42), younger age (2.72, 2.03–3.65), lower education level (1.32, 1.02–1.71), and greater severity of depression (4.94, 3.58–6.82).

**Conclusion:**

Factors that contributed to undetermined attitudes included male gender, younger age, lower education level, greater severity of depression, and lack of personal experience of an acquaintance's suicide. Passive thoughts regarding suicide prevention and the promotion of mental health were associated with male gender, younger age, lower education level, and greater severity of depression.

## Background

Grieving the loss of a family member or friend is a normal process. However, some grief reactions are subject to complications that may seriously compromise the health of the bereaved. For example, the loss of a close relative or friend by sudden traumatic death or suicide can complicate grieving. Farberrow et al. [1] suggested that the process of bereavement is more difficult for those bereaved by a suicide, especially during the first year, as compared to those bereaved by a death from natural causes.

Complicated grief may increase the risk of intense and prolonged mourning, depressive or anxiety disorders, and poor physical health [2,3]. In particular, suicide represents a factor that contributes to the complicated grief reactions of those affected, and complicated grief reactions may place such bereaved persons at risk for long-term dysfunction. Latham et al. [4] suggested that complicated grief is an independent psychiatric risk factor for suicidal thoughts and actions. In fact, the suicide of a spouse increases the suicide risk of the surviving spouse [5]. For these reasons, it is critical that appropriate support is available for individuals who have lost a loved one to suicide.

Early studies examining the effects of post-suicide support on grieving family members focused on professional interventions (i.e., interventions by physicians, nurses, and therapists) by gathering data from the bereaved [6-10]. It is widely believed that earlier post-suicide interventions are correlated with better outcomes [11]. However, there has been a notable absence of population-based studies examining attitudes towards those grieving a death by suicide.

Two studies examined the responses of college students to acquaintances grieving a death by suicide [12,13]. According to one study, respondents experienced greater difficulty talking with and expressing sympathy for family members bereaved by suicide as compared to family members bereaved by a death that was accidental or from natural causes. The other study reported that students tended to attribute more blame to those bereaved by a death by suicide than to those grieving a death resulting from other causes. David et al. [14] suggested that the self-blame and guilt that already haunt those dealing with the suicide of a loved one may receive reinforcement from their community. In view of the aforementioned findings that those grieving a death by suicide already face higher risks for complicated grief, it becomes clear that social reinforcement of the self-blame and guilt experienced by those bereaved by suicide requires replacement with community attitudes that reflect knowledge about suicide and compassion for the bereaved.

Despite the importance of this issue, we know of no study that has examined the factors that contribute to community (e.g., neighbours, workmates, and friends) attitudes towards people bereaved by the suicide of a friend or family member. However, the development of effective educational approaches requires relevant data. Therefore, we examined attitudes towards those bereaved by suicide and perceptions of suicide prevention and the promotion of mental health in a rural region of Japan.

## Methods

### Sample and procedure

We used a sample drawn from a rural town in Akita Prefecture, located in northern Japan. Annual mortality rates for deaths attributed to suicide in Japan and in Akita Prefecture in 2004 were 23.8 (males: 35.2; females: 12.8) and 42.1 (males: 65.7; females: 20.7) per 100,000 people, respectively. Since 1995, Akita Prefecture has recorded the highest suicide-related mortality rates in Japan. In 2004, the town that we studied recorded a suicide-related mortality rate of 47.3 per 100,000. Since 2006, this town has organised lectures on suicide and encouraged appointments at mental health counselling services to prevent suicide and promote mental health.

We conducted a survey from July through September 2005 using community volunteers to distribute questionnaires to all households and then collect them 2 weeks later. Our target population consisted of town residents aged 30 to 69 (institutionalised residents were excluded). Of 23,815 total residents, 7521 met these screening criteria. A total of 6383 residents responded to this survey (i.e., a response rate of 84.9%).

### Questionnaire

The questionnaire obtained data on demographic variables, educational history, and issues related to suicide. The latter included personal experience of an acquaintance's suicide (i.e., "Did you personally know anyone who died by committing suicide?"); attitudes towards those bereaved by a suicide (i.e., "If someone you know lost a family member or a close friend by suicide, how would you react to them?" The options were: offer to talk, watch calmly, not want involvement, or "don't know"); and perceptions regarding suicide prevention and the promotion of mental health in the community (i.e., "In your opinion, what is the attitude of your community towards efforts to prevent suicide and improve mental health?" The options were: "favourable," "unfavourable," or "don't know").

Educational background was classified into three levels: completed compulsory education (9 years or less); high school graduate (10–12 years); junior college graduate, vocational school graduate, or other higher education (more than 13 years).

The severity of depression was assessed using the Zung self-rating depression scale (SDS) [15]. Respondents were classified into four levels: normal (a raw score of < 40); mild (a raw score of 40–47); moderate (a raw score of 48–55); and severe (a raw score of ≥ 56).

### Statistical Analysis

Data on attitudes towards those bereaved by a suicide were classified into three groups of appropriate, undetermined, and inappropriate: offer to talk and watch calmly were classified as indicators of appropriate attitudes, "don't know" was undetermined, and "not want involvement" was inappropriate. Perceptions regarding suicide prevention and the promotion of mental health in the community were classified into two groups: responses representing favourable opinions were classified as "positive" thoughts, whereas responses representing unfavourable opinions or "don't know" were classified as "passive" thoughts. Logistic regression analyses identified factors contributing to undetermined attitudes and inappropriate attitudes toward the bereaved and compared these with appropriate attitudes and to passive thoughts regarding suicide prevention and the promotion of mental health in the community. For all statistical tests, two-tailed tests were used to determine statistical significance at P < 0.05. Statistical analyses were conducted using SPSS version 11.5 software (SPSS Inc.).

This survey was approved by the Ethics Committee of the School of Medicine at Akita University.

## Results

Data were obtained from respondents regarding the key areas under study (Table 1). Of the 6383 community residents who returned questionnaires, 5154 (80.7%) gave complete answers for all variables included in the logistic regression analysis (Table 1). Data on the prevalence of depression indicated that 19.4% of the total sample reported moderate depression and 4.1% reported severe depression.

Half of all respondents indicted that at least one of their acquaintances had committed suicide. Slightly more than two-thirds of respondents demonstrated "appropriate" attitudes towards those bereaved by a suicide (i.e., 23.0% would offer to talk and 44.5% would watch calmly), 30.4% demonstrated "undetermined" attitudes, and 2.1% demonstrated "inappropriate" attitudes. Overall, 16.0% demonstrated passive thoughts about suicide prevention and the promotion of mental health in the community.

**Table 1:** Characteristics of study participants.

Variable	Subgroup	% (N = 5154)
**Age (years)**		
	30–39	18.7
	40–49	27.4
	50–59	32.5
	60–69	21.4
**Gender**		
	Male	47.9
	Female	52.1
**Educational background (years)**		
	9 or less	23.3
	10–12	55.2
	13 or more	21.5
**Self-rated depression scale**		
	Normal	35.3
	Mild	41.2
	Moderate	19.4
	Severe	4.1
**Personal experience of an acquaintance's suicide**		
	Yes	52.6
	No	47.4
**Attitude toward those bereaved by a suicide**		
	Offer to talk	23.0
	Watch calmly	44.5
	Do not want to be involved	2.1
	Do not know	30.4
**Perceptions regarding suicide prevention and the promotion of mental health in the community**		
	Favorable	84.0
	Unfavorable	1.1
	Do not know	14.9

Table 2 presents the results of the logistic regression analyses, comparing "appropriate" attitudes towards those bereaved by a suicide, "undetermined" attitudes associated with male gender (adjusted OR 1.42, 95%CI = 1.26–1.61), younger age (2.64, 2.12–3.29), lower education level (1.32, 1.07–1.62), greater severity of depression (3.81, 2.80–5.20), and lack of personal experience of an acquaintance's suicide (1.39, 1.22–1.57). "Inappropriate" attitudes were associated with male gender (adjusted OR 1.98, 95%CI = 1.33–2.94), lower education level (2.55, 1.34–4.83), and greater severity of depression (6.93, 3.52–13.67). Both "undetermined" and "inappropriate" attitudes were related to male gender, lower education level, and greater severity of depression.

**Table 2:** Multiple logistic analysis for attitudes toward those bereaved by a suicide.

	Attitude toward those bereaved by a suicide				Perceptions of suicide prevention	
	Undetermined attitudes (N = 1568)/Appropriate attitudes		Inappropriate attitudes (N = 108)/Appropriate attitudes		Passive thoughts (N = 824)/Positive thoughts	
Variable	Adjusted OR	95%CI	Adjusted OR	95%CI	Adjusted OR	95%CI
Age (years)						
30–39	2.64	2.12–3.29	1.11	0.53–2.33	2.72	2.03–3.65
40–49	1.84	1.49–2.27	1.21	0.64–2.30	2.20	1.65–2.92
50–59	1.49	1.23–1.80	1.25	0.74–2.12	2.05	1.58–2.66
60–69	Reference		Reference		Reference	
Gender						
Male	1.42	1.26–1.61	1.98	1.33–2.94	1.22	1.04–1.42
Female	Reference		Reference		Reference	
Educational background (years)						
9 or less	1.32	1.07–1.62	2.55	1.34–4.83	1.32	1.02–1.71
10–12	1.30	1.11–1.52	1.18	0.67–2.08	1.10	0.90–1.34
13 or more	Reference		Reference		Reference	
Self-rated depression scale						
Normal	Reference		Reference		Reference	
Mild	1.33	1.15–1.54	0.79	0.48–1.28	1.40	1.15–1.70
Moderate	2.00	1.68–2.38	1.69	1.00–2.87	2.40	1.94–2.98
Severe	3.81	2.80–5.20	6.93	3.52–13.67	4.94	3.58–6.82
Personal experience of an acquaintance's suicide						
Yes	Reference		Reference		Reference	
No	1.39	1.22–1.57	1.03	0.69–1.54	1.13	0.97–1.32

Multiple logistic regression models were adjusted for all items listed in the table.

OR: Odds ratio CI: Confidence interval

Passive thoughts regarding suicide prevention and the promotion of mental health in the community were associated with male gender (adjusted OR 1.22, 95%CI = 1.04–1.42), younger age (2.72, 2.03–3.65), lower education level (1.32, 1.02–1.71), and greater severity of depression (4.94, 3.58–6.82).

## Discussion

Factors that contributed to "undetermined" attitudes included male gender, younger age, lower education level, greater severity of depression, and lack of personal experience of an acquaintance's suicide. Passive thoughts regarding suicide prevention and the promotion of mental health in the community were significantly associated with male gender, younger age, lower education level and greater severity of depression.

Many of the studies have reported that female gender is associated with a higher prevalence of poor mental health status than male [16-24]. Vahtera et al. [20] suggested that women are more affected by the aftermath of death or illness in their extended family than are men. Two studies have found that women tend to complain about stressful interpersonal events, whereas men tend to complain about stressful legal and work-related events [21,22]. Another review, however, suggested that men were more vulnerable to stressful events such as the loss of their partner [25]. Gender differences in the vulnerability to stressful life events were uncertain. Our results indicated that women would feel deeper sympathy than men for those mourning a death by suicide and might be more sensitive to bereaved individuals. Determining effective social support from within the community for individuals grieving the suicide of a friend or family member will require a consideration of gender differences.

Younger people tended to show "undetermined" attitudes towards those bereaved by suicide more than older people did. We considered the possibility that this association may be due to the relative inexperience of young people with bereavement. Although there are no data on the relationship between age and attitudes towards those bereaved by a suicide, studies of the relationship between age and worry (i.e., as assessed by Worry Scale or the Penn State Worry Questionnaire) show that older adults have reported low levels of worrying [26,27]. Powers et al. reported that the elderly worry significantly less than those who are relatively young [28]. These authors interpreted these results in terms of lifecycle span and experience. Our results support their findings.

The correlation between passive thoughts regarding suicide prevention and the promotion of mental health in the community and younger age may be understood as a result of disinterest owing to a relative lack of experience of young people with death from any cause.

Many studies have reported significant correlations between level of education and mental health status [23,24,29-31]. Wolff et al. [32] reported that attitudes of goodwill towards people suffering from mental illness were significantly associated with higher levels of education. It is possible that people with lower levels of education express unhelpful attitudes towards those grieving the suicide of a loved one because of a general ignorance about mental health issues. For example, Kaneko et al. [33] reported that low mental health literacy was strongly associated with low levels of education. People with low levels of education may display the undetermined or inappropriate attitudes reported here because they know less about suicide and mental illness.

Beck et al. [34] proposed that depression emerges from dysfunctional cognitions that initiate and maintain the emotional, motivational, and behavioural symptoms that define this condition (e.g., dysphoric mood, lack of motivation, and low energy level). Additionally, the features of depression include reduced energy and diminished activity [35]. These symptoms of depression are consistent with our finding of an association between inappropriate or undetermined attitudes, and greater severity of depression.

Personal experience, or the lack thereof, of involvement with individuals who commit suicide appears to affect attitudes towards those grieving a death by suicide. Addison et al. [36] reported that people who had personal experience of people suffering from mental illness were generally more positive in their attitudes towards people with mental illness compared to those who had no such experience. Our results support the notion that personal experience affects attitudes towards others who are similarly situated. Harwood et al. [37] reported that relatives and friends grieving a death by suicide scored higher than those grieving deaths by other causes in measurements of stigma, shame, sense of rejection, and unique reactions. They found that bereavement following suicide affects not only family but also friends and neighbours. This may explain our finding that appropriate attitudes in the form of expressions of feelings for those mourning a death by suicide is related to a history of personal contact with someone who commits suicide as compared with undetermined attitudes.

Those who displayed undetermined and inappropriate attitudes towards those bereaved by a suicide tended to focus less on the bereaved persons than did those who displayed appropriate attitudes. We categorised "offering to talk" as an appropriate attitude. However, those who indicted they would offer to talk also require education about appropriate ways of talking, language use and grief procedures. Further studies will be needed to improve the social support for those bereaved by suicide and to reduce their psychological burden.

Several limitations pertaining to our study should be noted. Because we used a cross-sectional design, we could not determine causal relationships. Second, the use of a self-administered questionnaire may have led to reporting bias, perhaps especially in relation to items about personal experience of an acquaintance's suicide. Although this may have had a role in the responses proffered by older subjects, similar results were obtained when the sample was stratified by age. Third, our study did not include other causes of death such as natural or accidental death. However, some studies have indicated that the social response to death by suicide differs from responses to other causes of death including natural death [12,13]. Therefore, we consider that our results sufficiently should reflect attitudes toward those bereaved by a suicide. Strategies for improving attitudes and perceptions within the social network of an individual who is bereaved by a suicide should be helpful for the individual afflicted by grief.

Of the 6383 respondents, 5154 answered all of the items used in the logistic regression analyses. The analyses included 68.5% of residents in the survey area aged 30–69 years. The percentage of unanswered questions were respectively as follows: gender, 2.0%; age, 2.6%; level of education, 1.7%; level of depression, 13.1%; and personal experience of an acquaintance's suicide, 3.4%. Of the 1229 respondents who did not complete the questionnaire, 41.6% were male, 10.6% were aged in their 30s, 12.8% had an educational background of 13 or more years, 30.3% had no depressive symptoms (normal) based on a self-rated depression scale, and 41.9% had an acquaintance who had committed suicide. Males, younger people, those with higher levels of education, those with no depressive symptoms, and those who had personal experience of an acquaintance's suicide returned questionnaires with significantly more complete answers than did others. No statistically significant differences with respect to attitudes towards those bereaved by suicide appeared between complete responders and incomplete responders. However, a statistically significant difference appeared between complete and incomplete responders with regard to perceptions regarding suicide prevention and mental health promotion the community. Levels of concern about suicide among the residents questioned may have influenced these differences.

The community of the area that we studied experiences many deaths by suicide. Prevention of suicides is needed. In developing effective community suicide prevention programs, our results should be taken into account and utilised to educate the community regarding appropriate attitudes and actions.

## Conclusion

Undetermined attitudes towards those bereaved by a suicide were associated with male gender, younger age, lower education level, greater severity of depression, and lack of personal experience of an acquaintance's suicide, as compared to those with appropriate attitudes. Passive thoughts regarding suicide prevention and the promotion of mental health in the community were significantly associated with male gender, younger age, lower education level and greater severity of depression. We hope that our findings will raise awareness of the complications affecting bereavement after a suicide and will improve efforts to help those engaged in the bereavement process. Further research will be required to evaluate the effectiveness of adequate intervention.

## Competing interests

The authors declare that they have no competing interests.

## Authors' contributions

SM, YM, and YK made substantial contributions to the conception and design of the study and were involved in drafting and reviewing the manuscript. MS and MY contributed to the data acquisition process. SM, YM, and YK contributed to the analysis and interpretation of the data. All authors have read and approved the final manuscript.

## Pre-publication history

The pre-publication history for this paper can be accessed here:


